# Arbovirus Seropositivity in UK Zoo-Housed Birds, with Evidence for Autochthonous Exposure to Batai Virus

**DOI:** 10.3390/microorganisms14081687

**Published:** 2026-08-01

**Authors:** Karen L. Mansfield, Insiyah Parekh, Estela Gonzalez, Arran J. Folly, Ethan Wrigglesworth, Amanda Guthrie, Sheryl Bradley, Simon Spiro, Nicholas Johnson

**Affiliations:** 1 Animal and Plant Health Agency, Woodham Lane, New Haw KT15 3NB, UK; insiyah.parekh@apha.gov.uk (I.P.); estela.gonzalezfernandez@apha.gov.uk (E.G.); arran.folly@apha.gov.uk (A.J.F.); nick.johnson@apha.gov.uk (N.J.); 2 Wildlife Health Services, Zoological Society of London, London NW1 4RY, UK; ethan.wrigglesworth@zsl.org (E.W.); amanda.guthrie@zsl.org (A.G.); sheryl.bradley@zsl.org (S.B.); simon.spiro@zsl.org (S.S.); 3 Faculty of Health and Medical Sciences, University of Surrey, Guildford GU2 7XH, UK

**Keywords:** zoo, bird, antibody, Batai virus, orthoflavivirus

## Abstract

Mosquito-borne virus transmission continues to increase in Europe, with the emergence of Usutu virus (USUV) in the United Kingdom (UK) in 2020 and the geographical expansion of West Nile virus (WNV) to more northerly latitudes. Other mosquito-borne viruses also circulate throughout mainland Europe, including Batai virus (BATV), leading to seropositivity in a range of vertebrate species, including humans, livestock and birds. A panel of archived sera from birds housed within a zoological collection in Central London was investigated for antibodies raised against the mosquito-borne viruses USUV, WNV and BATV. Orthoflavivirus seropositivity was detected in several birds, along with antibodies raised against BATV, an orthobunyavirus previously not reported in the UK. Furthermore, seropositivity in birds bred in captivity provides evidence of cryptic transmission of mosquito-borne viruses. This study highlights the utility of zoological collection birds as sentinels for VBD detection.

## 1. Introduction

Vector-borne diseases (VBDs) continue to expand in geographical range throughout Europe, fuelled in part by the impact of changing climate on arthropod vector populations [1,2]. This includes reports of the mosquito-borne orthoflaviviruses Usutu virus (USUV) and West Nile virus (WNV) in regions where they were previously undetected [3,4]. Both viruses exist in transmission cycles involving birds [5,6,7], and are now considered endemic throughout much of mainland Europe [8]. Migratory bird movements are likely to drive introduction of arthropod-borne viruses [9,10,11,12]. Both viruses are considered zoonotic [13], with spillover into dead-end hosts such as horses and humans (reviewed in [14]). In horses, WNV infection can cause severe neurological disease, whereas USUV infections are generally asymptomatic [15]. However, the involvement of WNV in human disease is well-known, causing potentially fatal neuroinvasive disease in a small number of cases [16]. Although fewer human USUV cases are reported, these can range from being asymptomatic to meningitis or meningoencephalitis which again can be fatal [17].

In the UK, USUV was first detected at a London zoological park in 2020, in local resident wild blackbirds and sparrows, with subsequent positive USUV serology from zoological collection birds at the same site, indicating sustained transmission [18,19]. Additionally, WNV RNA was detected in retrospective testing of a pool of UK *Aedes vexans* mosquitoes, collected in 2023 [20]. Alongside USUV and WNV, several other mosquito-borne viruses circulate in Europe, including the orthobunyavirus Batai virus (BATV) [21,22,23,24], which has not been previously reported in the UK. BATV also circulates in wild birds, and is generally asymptomatic in domestic cattle (reviewed in [21]), although mild illness was reported in sheep and goats in India due to the ‘Chittoor’ strain [25]. BATV has limited zoonotic impact [24] with rare human infections generally manifesting as mild influenza-like symptoms [26].

Following the initial detection of USUV in zoological collection birds at ZSL London Zoo in 2020, sero-surveillance efforts continue to regularly monitor the detection of anti-orthoflavivirus antibodies in avian sera at the site. However, a subset of samples were investigated to assess seropositivity against both orthoflaviviruses and BATV, to determine whether cryptic transmission of BATV is occurring within avian reservoirs in the UK, and to provide further evidence for the utility of zoo-housed birds as sentinels for arbovirus detection.

## 2. Materials and Methods

A panel of archived sera from a zoological collection in Central London (Zoological Society of London [ZSL]) was investigated for antibodies against USUV, WNV and BATV, to identify seropositivity and inform on the utility of zoological collection birds as sentinels for VBD detection. This study contributes to the ongoing orthoflavivirus antibody surveillance in birds housed at this site [18,19], where a discrete subset of sera were additionally assessed for antibodies raised against BATV. Archived serum samples (*n* = 63) from healthy birds (*n* = 54) housed at ZSL London Zoo were retrieved. These samples were originally collected by veterinarians during routine preventive care or interventional examinations; no animals were sampled specifically for this study or any other study. Sera included in the study encompassed a range of species within 12 avian orders (*Accipitriformes*, *Anseriformes*, *Bucerotiformes*, *Charadriiformes*, *Ciconiiformes*, *Falconiformes*, *Galliformes*, *Gruiformes*, *Passeriformes*, *Pelecaniformes*, *Psittaciformes*, *Strigiformes*).

To retrospectively assess archived sera for antibody presence, heat-inactivated sera were screened for anti-orthoflavivirus antibodies using the ID Screen^®^ Flavivirus Competition ELISA (Innovative Diagnostics, Grabels, France) according to manufacturer instructions. Samples yielding a positive or a doubtful ELISA result were further assessed by plaque reduction neutralisation test (PRNT) for WNV- and USUV-specific neutralising antibodies using the methodology as previously described [27]. Briefly, serum dilutions (2-fold, 1:10 to 1:1280) were incubated with virus strains WNV Goose Israel 1998 (lineage 1) (provided by the Kimron Veterinary Institute, Israel), WNV 291.B/2013/Velky Biel/SVK (lineage 2), or USUV (XT-614-20/27-8-2020) for 60 min at 37 °C/5% CO_2_, prior to incubation with Vero C1008 cells (1 day-old cultures in 12-well plates) overlayed with carboxymethylcellulose (CMC) overlay. The overlay contained 7.5 g CMC, 1X Minimum Essential Medium Eagle with Earle’s salts, 1% foetal bovine serum, 0.2% sodium bicarbonate, 0.02 M HEPES ((4-(2-hydroxyethyl)-1-piperazineethanesulfonic acid), 2 mM L-glutamine and penicillin/streptomycin (all components sourced from Sigma Aldrich, Merck Life Sciences UK Limited, Gillingham, UK), After incubation for 6 days, infected cell monolayers were fixed with 10% neutral buffered formalin (NBF) and stained with 2.3% crystal violet (Sigma Aldrich, Merck Life Sciences UK Limited, Gillingham, UK) to visualise the plaques for counting. Additionally, all sera were tested by PRNT for anti-BATV antibodies using BATV strain 53.2. PRNT titres were calculated in relation to the mean plaque count from triplicate virus control wells on each plate, and were reported as PRNT_90_, a stringent assessment of antibody titre reflecting serum dilutions at which 90% virus neutralisation occurred; hence a PRNT_90_ titre of 1:10 is considered seropositive. Determination of 95% confidence intervals (CI) for the prevalence percentages (proportions) were undertaken in GraphPad Prism version 8.4.2 (GraphPad Software, San Diego, CA, USA).

## 3. Results

In total, 13/63 serum samples (reflecting 10/54 individual birds) yielded positive antibody detections against USUV, WNV and/or BATV.

### 3.1. Detection of Anti-Orthoflavivirus Antibodies

In terms of anti-orthoflavivirus antibodies, seroprevalence at the individual bird level for anti-USUV antibodies was 14.8% (95% CI: 7.7–26.6%) (Table 1).

Antibody titres against USUV (between 1:10 and 1:80 at PRNT_90_) were obtained from eight birds (Abdim’s storks [*Ciconia abdimii*, *n* = 4], black-necked stilt [*Himantopus mexicanus*], Javan green magpie [*Cissa thalassina*], barn owl [*Tyto alba*] and great white pelican [*Pelecanus onocrotalus*]) in samples taken between 2021 and 2023 (Table 2). The Abdim’s storks and barn owl were bred in captivity in the UK, suggesting that USUV exposure occurred in the UK, providing further evidence for the continued circulation of USUV at this site since emergence in 2020. Seroprevalence of anti-WNV antibodies (lineage 1 or lineage 2) was lower at 1.9% (95% CI: 0.1–9.8%) (Table 1), reflecting three samples from a single great white pelican with high antibody titres (ranging from 1:80 to 1:160 in duplicate independent assays) against both USUV and WNV at PRNT_90_ (Table 2), with anti-WNV antibodies detected in all three samples from this bird in two independent assays. The circumstances of WNV exposure are unknown, since the country of origin and full history for this long-lived bird is unclear. However, the high PRNT_90_ titres for both anti-USUV and anti-WNV antibodies, along with the lack of anti-USUV antibodies in the first sample (October 2020), suggest that this bird was historically exposed to WNV (at location unknown) and then subsequently exposed to USUV whilst at the zoo between October 2020 (first sample) and December 2022 (second sample). A third sample (March 2025) from this bird confirmed the persistence of antibodies against both WNV and USUV. However, it can be challenging to definitively differentiate between USUV and WNV as the viral source of these antibodies, due to the inherent cross-neutralisation between orthoflaviviruses [27].

### 3.2. Detection of Anti-BATV Antibodies

Assessment for anti-BATV antibodies identified the first detection in UK birds, where three samples (3/63, 4.8%) yielded antibody titres at PRNT_90_, reflecting a seroprevalence in 3/54 birds of 5.5% (95% CI: 1.5–15.1%) (Table 1). Seropositive birds included Von der Decken’s hornbill (*Tockus deckeni*), woolly-necked stork (*Ciconia episcopus*) and Javan green magpie (Table 3).

The Von der Decken’s hornbill was bred in captivity in the UK, suggesting that BATV exposure occurred in the UK prior to blood sampling in May 2021, either at the zoo or elsewhere in the UK. There was a discrepancy between PRNT_90_ titres for this sample, and due to the small sample volume, it was not possible to complete further assessment. Despite this, the 1:10 titre does indicate seroconversion, albeit at a low titre. One serum sample from the Javan green magpie also contained anti-USUV antibodies (Table 2); in terms of the detection of antibodies against both USUV and BATV in the same sample, USUV and BATV belong to different virus families (*Flaviviridae* and *Peribunyaviridae*, respectively), with little or no antigenic similarity, hence cross-neutralisation between these two distantly related viruses would be unlikely. This implies the genuine detection of antibodies against both viruses in the same sample, differentiated sufficiently by plaque assay and related to historical virus exposure. Although the origin of this bird is non-UK (Czechia), this sample was the second sample taken at the zoo for this bird (late May 2023). The timing of the two samples, along with the detection of antibodies against only USUV in the first sample (early May 2023), suggests that exposure to BATV occurred within a discrete window of time during May 2023 whilst the bird was resident at the zoo. This provides evidence for multiple transmission events of BATV, firstly before May 2021 (Von der Decken’s hornbill) at a UK location (at the zoo, or elsewhere), and again in the middle of May 2023 (Javan green magpie) at the zoo.

## 4. Discussion

We report the continued detection of antibodies raised against orthoflaviviruses in sera from collection birds housed at ZSL London Zoo, along with the first report of antibodies raised against the orthobunyavirus BATV through retrospective testing. The PRNT_90_ provides a robust antibody titre as evidence of prior infection, although antibody levels were variable. The majority of sera tested originated from birds with unknown or non-UK origin, although positive serology from several birds bred in captivity in the UK provided evidence for autochthonous exposure to USUV and BATV, presumably from mosquito bites, either at ZSL or elsewhere in the UK. However, as some birds had resided at other locations prior to ZSL London Zoo, it may be difficult to identify the timing and location of virus exposure. The zoological collection birds assessed in this study are housed in outdoor enclosures that are likely to be accessible to ornithophilic mosquitoes. Additionally, a broad range of mosquito species has been implicated in BATV transmission in Europe, predominantly *Anopheles maculipennis s.l.* [28], but also *Culiseta* sp., *Aedes vexans*, *Aedes detritus*, *Culex pipiens s.l.* and *Culex modestus* [21], and several of these species are detected in the vicinity of the zoo [29]. However, mosquito surveillance at the site between 2022 and 2024 did not detect any BATV genome [29], hence prevalence in local mosquitoes is likely to be extremely low. However, European sero-surveillance reveals increasing evidence of seroconversion to BATV in livestock and wild birds [21,23,30]; hence, this first detection of anti-BATV antibodies in UK birds is not unsurprising. Although the number of samples assessed in this study is relatively limited, these data, along with the continued detection of USUV in wild UK birds [18,19], highlight the involvement of birds in many arbovirus transmission cycles, often occurring cryptically. Overall, these findings underscore the utility of birds housed in zoological collections as sentinels for arbovirus surveillance [26,31,32], and sero-surveillance will continue at this site in London to gain further insights into the involvement of zoological collection birds in the transmission of mosquito-borne viruses.

## Figures and Tables

**Table 1:** Seropositivity prevalence estimation for antibodies raised against USUV, WNV and BATV in archived serum samples (*n* = 63) from zoological collection birds (*n* = 54), determined by PRNT_90_. The Wilson/Brown method (Parts of Whole data) was applied to determine 95% confidence intervals (CI) for prevalence percentages (proportions) in GraphPad Prism 8.4.2.

Virus	Seropositivity at the Individual Bird Level (*n* = 54)		
	Number of Seropositive Birds	Prevalence (%)	95% CI
Usutu virus	8	14.8	7.7–26.6
West Nile virus (lineage 1)	1	1.9	0.1–9.8
West Nile virus (lineage 2)	1	1.9	0.1–9.8
Batai virus	3	5.5	1.5–15.1

**Table 2:** Retrospective detection of antibodies raised against orthoflaviviruses in zoological collection birds, determined by orthoflavivirus ELISA and PRNT_90_. The PRNT_90_ results are derived from duplicate independent assays, where results vary both titres are shown. Positive results from birds born in captivity (BIC) in the UK are shown in bold type.

Species (Common)	Species (Latin)	Country of Origin	Sampling Date	Orthoflavivirus ELISA	USUV PRNT_90_	WNV Lineage 2 PRNT_90_	WNV Lineage 1 PRNT_90_
Abdim’s stork #1	*Ciconia abdimii*	BIC (ZSL London Zoo)	25 October 2021	**Positive**	**1:40**	Negative	ND
Abdim’s stork #2	*Ciconia abdimii*	BIC (ZSL London Zoo)	22 November 2021	**Positive**	**1:40**	Negative	ND
Abdim’s stork #3	*Ciconia abdimii*	BIC (ZSL London Zoo)	13 December 2021	**Positive**	**1:40**	Negative	ND
Black-necked stilt	*Himantopus mexicanus*	Unknown	16 December 2021	Positive	1:10, 1:20	Negative	ND
Barn owl	*Tyto alba*	BIC (England, Essex)	6 January 2022	**Positive**	**1:10**	Negative	ND
Abdim’s stork #4	*Ciconia abdimii*	BIC (ZSL London Zoo)	9 December 2022	**Doubtful**	**1:20**	Negative	ND
Javan green magpie	*Cissa thalassina*	BIC (Czechia)	5 May 2023	Positive	1:10, 1:20	Negative	ND
			22 May 2023 *	Positive	1:10	Negative	ND
Great white pelican	*Pelecanus onocrotalus*	Unknown	5 October 2020	Positive	Negative	1:80	1:80
			9 December 2022	Positive	1:80	1:80, 1:160	1:80
			17 March 2025	ND	1:40	1:80	1:80

* This sample also contained antibodies against BATV. BIC, born in captivity, ND, not done.

**Table 3:** Retrospective detection of antibodies raised against BATV in zoological collection birds, determined by PRNT. The PRNT_90_ results are derived from duplicate independent assays, where the results vary both titres are shown. Positive results from birds born in captivity (BIC) in the UK are shown in bold type.

Species (Common)	Species (Latin)	Country of Origin	Sampling Date	BATV PRNT_90_
Von der Decken’s hornbill	*Tockus deckeni*	BIC (England, Cornwall)	12 May 2021	**1:10, 1:80**
Woolly-necked stork	*Ciconia episcopus*	Born in the wild (India)	16 February 2021	1:10, 1:20
Javan green magpie	*Cissa thalassina*	BIC (Czechia)	5 May 2023	Negative
			22 May 2023 *	1:10

* This sample also contained antibodies against USUV. BIC, born in captivity.

## Data Availability

The original contributions presented in this study are included in the article. Further inquiries can be directed to the corresponding author.

## Abbreviations

The following abbreviations are used in this manuscript:

BATV	Batai virus
BIC	Born in captivity
CI	Confidence interval
CMC	Carboxymethylcellulose
NBF	Neutral buffered formalin
PRNT	Plaque reduction neutralisation test
UK	United Kingdom
USUV	Usutu virus
WNV	West Nile virus
ZSL	Zoological Society of London
